# Naloxone prescription fills and use by patients treated for opioid use disorder by telehealth

**DOI:** 10.1016/j.dadr.2024.100244

**Published:** 2024-05-28

**Authors:** Scott G. Weiner, Emily N. Miller, Barbara Burke, Brian Clear

**Affiliations:** a Bicycle Health, Boston, MA, USA; b Department of Emergency Medicine, Brigham and Women’s Hospital, , Massachusetts, Boston, MA, USA

**Keywords:** Naloxone, Opioid use disorder, Harm reduction

## Abstract

**Background:**

It is unknown how many people in treatment for opioid use disorder (OUD) have naloxone, use naloxone, and what their perceptions and barriers to obtaining it are.

**Methods:**

This was a survey of patients treated in a large telehealth OUD program. Between December 6, 2023 and January 6, 2024, all patients who had access to the program’s phone app (n=17,899 individuals, of whom 12,887 were in active treatment), were invited to complete an anonymous online survey.

**Results:**

There were 701 individuals who completed the survey. Nearly all patients (n=693, 99%) knew what naloxone is, and the majority (n=601, 86%) knew how to administer it. A quarter of these patients (n=177, 25%) reported either having naloxone used on themselves or using it on someone else. 161 patients (23%) reported taking a naloxone training course. Of patients who recalled receiving a prescription, 72% (n=382) filled the prescription, and 85% (n=321) reported that insurance paid for all or part of it. If filled, the naloxone was reported as used by 30 (8%) patients. If not filled, reasons were: already had it (n=55, 37%), did not think it was needed (n=54, 37%) or too expensive (n=36, 23%). Patients who reported knowing how to administer naloxone (OR 2.63 (95% CI 1.35–5.00) were more likely to fill the prescription.

**Conclusions:**

Patients prescribed naloxone in a telehealth treatment program filled the prescription 72% of the time, and when it was filled, 8% used the naloxone. Education and cost policy changes may reduce barriers to obtaining naloxone.

## Highlights

• Naloxone co-prescription is recommended for patients with opioid use disorder.
• Of 701 patients treated in a telehealth program, 72% filled the naloxone prescription.
• When filled, naloxone was used by 8% of patients on themselves or another person.
• Barriers to obtaining naloxone included cost and thinking it not was needed.

## 1 Introduction

The opioid overdose epidemic continues to burden affect communities around the world. In the U.S. specifically, between 1999 and 2021, nearly 650,000 people died from opioid overdoses (Centers for Disease Control and Prevention, n.d.). Overdose deaths have increased substantially in the U.S. over this time frame; the number of overdose deaths in 2021 was ten times the number in 1999 (Centers for Disease Control and Prevention, n.d.). Opioid overdoses can be reversed by using the drug, naloxone. Naloxone is a competitive antagonist at the mu-opioid receptor that can be administered by many routes, including by bystanders and lay public via nasal spray (Taylor & Lasser, 2024). The Centers for Disease Control and Prevention reported that, in 2019, a bystander was present in nearly 40% of opioid and stimulant overdose deaths (O'Donnell et al., 2020). Having access to naloxone, as well as knowledge of how to administer it, can potentially save the life of someone experiencing an opioid overdose.

Best practices recommend that patients at risk for overdose, including those in treatment for opioid use disorder (OUD), have naloxone on hand in the case of return to useor if someone near to the person in treatment overdoses (U.S. Department of Health and Human Services, 2018). Likewise, ten states that mandated naloxone co-prescribing/offering saw a 16% reduction in the number of deaths related to prescription opioids (Sohn et al., 2023). Routinely co-prescribing naloxone alongside buprenorphine might also benefit the individual if there is return to drug use or if those around the individual experienceoverdose, as was demonstrated in a study showing that 18% of individuals enrolled in an opioid treatment program who were provided with education and take-home naloxone and reversed overdoses in their community (Katzman et al., 2020).

Existing literature indicates varying patient attitudes and practices regarding naloxone. One study found that while patient awareness of naloxone was high, there was also large variation in naloxone perceptions and practices, as some individuals treated for substance use disorder viewed it as unnecessary and others rated having it to be a priority (Heavey et al., 2018). Patients understand the rationale for naloxone prescribing but would benefit from education and awareness in order to foster positive perceptions of naloxone use (Mueller et al., 2017). Current gaps in knowledge include how often and why patients with OUD obtain naloxone, how often it is used, and the extent of barriers to obtaining naloxone such as insurance coverage and price.

In our practice, a large telehealth-only OUD treatment group, it is routine to co-prescribe a nasal naloxone rescue kit along with the first prescription for buprenorphine. However, it is not known at what rate patients fill their naloxone prescription, their knowledge about naloxone, their use of naloxone, and – if not filled – why not. Understanding why a patient with OUD does not fill their naloxone prescription can inform providers of strategies to increase patient understanding and motivation in having access to this potentially life-saving drug. This study surveyed a large sample of patients with OUD treated in the telehealth setting to ascertain their experiences with naloxone.

## 2 Material and methods

### 2.1 Setting and participants

This study was conducted in a telehealth treatment program. The program specializes in OUD treatment with buprenorphine, and sees patients exclusively by telehealth, with rare exceptions. To participate in treatment, patients need to be 18 years or older, have a safe place to store buprenorphine, have access to an internet-connected smartphone (assistance is provided to those who do not have this), and can fill prescriptions for buprenorphine at a pharmacy. The standardized first visit template includes a prescription for intranasal naloxone (with 9 refills). The prescriber can either send the prescription electronically to the patient’s preferred pharmacy or delete it if not desired.

The program utilizes a proprietary smartphone app that patients access for all aspects of their care, including video visits with their providers, therapists, peer recovery coaches, and to attend group meetings. Patients also use the app to schedule meetings and submit urine toxicology tests. The app includes a secure text messaging platform for communication with providers, other clinicians and support staff. At the time of this study, there were about 12,000 active patients and around 5,000 more who were no longer in treatment but still had access to the app. Roughly 40% of patients self-pay for the service (currently $199/month), and the remainder are covered by a range of insurances including commercial, Medicaid and Medicare. A small percentage of patients are justice-involved individuals who reside in Residential Reentry Centers (colloquially known as “halfway houses”) and their care is covered by a subcontract to the Federal Bureau of Prisons. All patients who had access to the app were included. There were no exclusion criteria. This study was approved by the Western-Copernicus Group (WCG) Institutional Review Board with waiver of informed consent by participants.

### 2.2 Survey methodology

This study utilized an anonymous survey methodology to query patients about naloxone. All patients who had access to the app were sent a text message that invited participants to complete a brief survey about naloxone. It was explicitly stated that the study was not part of their routine care, that individual results would be kept confidential, and that the responses would not be shared with their provider team. The message included a link to an online survey that included more formal consent language and then the survey instrument. After completion, participants could elect to enter a raffle for one of two $200 gift cards. The survey (Appendix 1) was only offered in English (similar to all other content on the app) and created by the research team without additional validation. The survey inquired about patients’ basic demographic information and included various questions related to naloxone. The primary outcomes of the study pertained to patient use and prescription filling of naloxone. This includes patient self-reported knowledge of naloxone, use of naloxone, and whether the patient has filled their prescription for naloxone. The survey had branch points, asking different questions, depending on if the patient filled our naloxone prescription or not. If they have not filled the prescription, they are asked to provide a reason for not filling it. Secondary outcomes examined were trends in naloxone self-reported knowledge, use, and prescription filling among different demographics, such as age, gender, race and ethnicity.

### 2.3 Data collection and analysis

The survey was active from December 6, 2023 to January 6, 2024. A one-month period was chosen as this is the maximum time expected between visits with a patient’s provider, and some patients only check their app messages at the time of their visit. Data were collected on the Jotform platform, exported to a spreadsheet, and analyzed using JMP v16.0 (SAS Institute, Cary, NC) for descriptive analysis. In addition, a multivariate logistic regression was used to estimate the odds of filling the naloxone prescription by patient-reported knowledge of how to use naloxone. Patients who reported receiving a prescription and did not already have naloxone were included in the analysis. One patient identified as non-binary and was excluded from the regression analysis (for instability of the gender variable due to small sample size). All variables were treated as dichotomous. Covariates included in the model were age (18–49 (reference) and 50 plus), gender (male (reference) and female), race (white (reference) and non-white), and ethnicity (not Hispanic or Latino (reference) and Hispanic or Latino). Logit coefficients were converted to odds ratios. Asymptotic standard errors were used to determine statistical significance of findings. Results were obtained using R version 4.1.2 with the lmtest package and glm() function (Zeileis et al., 2008; R Core Team, 2021). Although we initially planned to include the questions “do you know how to administer naloxone?”, “have you ever used naloxone?”, and “have you taken a naloxone training course?”, we discovered that they were collinear and thus could not independently predict the value of the dependent variable (filling the naloxone prescription). Therefore, we included “do you know how to administer naloxone?” as the representative question from this group to include as a predictor.

## 3 Results

The survey instrument was sent to 17,899 individuals, of whom 12,887 were considered active in treatment on the day the survey was sent out. A total of 701 surveys were completed. Demographic data are displayed on Table 1. Briefly, most patients who responded were aged 30–49 (n=451, 64%). There were more female patients than male (55% vs. 44%). Most patients were White and non-Hispanic/Latino (89%). Patients resided in 31 different states. About two-thirds of patients (69%) predominantly used prescription opioids before starting OUD treatment. There were 599 patients who reported their length in treatment with our group, which was median 10 (interquartile range (IQR) 5–21, range 1–69) months.

**Table 1:** Demographics and characteristics of survey respondents.

	n (%)
Age Range (years)	
18–29	71 (10.1%)
30–39	230 (32.8%)
40–49	221 (31.5%)
50–59	112 (16.0%)
60–69	53 (7.6%)
70 or older	14 (2.0%)
Gender	
female	386 (55.1%)
male	313 (44.7%)
non-binary	2 (0.2%)
Race	
American Indian/Alaska Native	19 (3.0%)
Asian	9 (1.3%)
Black or African American	43 (6.2%)
Native Hawaiian/Pacific Islander	7 (1.0%)
White	617 (88.8%)
Ethnicity	
Hispanic or Latino	75 (10.9%)
Not Hispanic or Latino	610 (89.1%)
State of primary residence	
Florida	98 (14.2%)
Texas	98 (14.2%)
California	90 (13.0%)
North Carolina	66 (9.5%)
Michigan	61 (8.8%)
Arizona	45 (6.5%)
Virginia	34 (4.9%)
Colorado	25 (3.6%)
Illinois	23 (3.3%)
Pennsylvania	20 (2.9%)
Other	132 (19.1%)
Primary Opioid Used	
prescription opioids	478 (68.6%)
fentanyl or heroin	203 (29.1%)
other	16 (2.3%)

Impressions and self-reported knowledge about naloxone are displayed on Table 2. Nearly all patients (99%) know what naloxone is, and the majority (86%) self-reported that they know how to administer it. A quarter of these patients (25%) reported either having naloxone used on themselves or using it on someone else. There were 161 patients (23%) who reported taking a naloxone training course. Even though it is routine practice to send a naloxone co-prescription along with a first buprenorphine prescription in our practice, 76% recalled receiving a prescription.

**Table 2:** Naloxone survey responses (N=701).

	n (%)
Do you know what naloxone is?	
Yes	693 (98.9%)
No	8 (1.1%)
Do you know how to administer naloxone?	
Yes	601 (85.9%)
No	99 (14.1%)
Have you ever used naloxone?	
Yes, on myself	23 (3.3%)
Yes, on someone else	125 (17.9%)
Yes, on both myself and someone else	29 (4.1%)
No	523 (74.7%)
Have you taken a naloxone training course?	
Yes	161 (23.0%)
No	540 (77.0%)
Did you receive a prescription for naloxone upon enrollment?	
Yes	533 (76.3%)
No	166 (23.7%)
Did you fill the prescription for naloxone?	
Yes	382 (71.7%)
No	151 (28.3%)
If not filled, why not?	
Too expensive	36 (23.3%)
Patient didn't think it was needed	54 (36.5%)
Already have it	55 (37.2%)
No pharmacy availability	3 (2.0%)
If filled, did insurance pay for part of it?	
Yes	321 (84.9%)
No	57 (15.1%)
If filled, was the naloxone used?	
Yes	30 (7.9%)
No	352 (92.1%)
Was the naloxone used for the patient or someone else?	
Patient	11 (35.5%)
Someone else	20 (64.5%)

The primary outcomes were to determine if patients a) filled a prescription for naloxone and b) if the naloxone was used either on themselves or someone else. There were 382 patients (54% of all patients, and 72% of the 533 who recalled receiving a prescription) who filled the prescription, and the naloxone was reported as used by 30 (8%) patients. Three hundred twenty-one patients (85%) reported that insurance paid for all or part of the prescription. Of these, 287 reported their co-pay amount: 172 (60%) had no co-pay. The remainder paid a median of $10 (IQR $7.47-$20). Fifty-seven patients reported that insurance did not pay for the naloxone. Of these, 42 reported their out-of-pocket cost. For 7 patients (12%) there was no out-of-pocket cost. The remainder paid a median of $60 (IQR $33.64-$95). There were many reasons why patients did not fill the prescription. Many patients already had it (n=55). Others didn’t think it was needed (n=54) or reported it was too expensive (n=36).

A multivariable logistic regression model was used to estimate the odds of patients filling their naloxone prescription (Table 3). The odds of filling the naloxone prescription was 163% higher among those who know how to administer naloxone compared to those who do not know how, keeping age, gender, ethnicity, and race constant. The odds of filling the prescription are elevated for patients who know how to administer naloxone (adjusted odds ratio 2.63, 95% confidence interval 1.35–5.00, p=0.004). Conversely, age, gender, race and ethnicity were not associated with variations in filling the prescription.

**Table 3:** Multivariate logistic regression determining which patient characteristics and answers were most likely to be associated with filling the naloxone prescription, excluding patients who reported that they already had naloxone.

	Odds ratio	95% confidence interval	p value*
Age			
18–49 years	reference		
50 years or older	1.25	0.71–2.29	0.46
Gender			
male	reference		
female	1.56	0.98–2.51	0.06
Race			
white		reference	
not white	1.82	0.83–4.61	0.17
Ethnicity			
Not Hispanic or Latino	reference		
Hispanic or Latino	1.39	0.65–3.36	0.43
Do you know how to administer naloxone?			
no	reference		
yes	2.63	1.35–5.00	**0.004**

* P value <0.05 is considered statistically significant and is in boldface.

## 4 Discussion

This study, a survey of patients who have been engaged in telehealth treatment for OUD with a large number of responses, describes several insights about patients’ understanding and experiences with naloxone that may inform future interventions. Almost all patients know what naloxone is, but about 14% reported that they did not know how to use it. This finding indicates an important gap and is similar to findings of a recent online survey of a community of people who use drugs that found that only 38% of participants who use opioids had received naloxone training, and that only 56% of these individuals said that they felt comfortable using a naloxone rescue kit (Schwieger et al., 2023). Naloxone training courses improve participants’ abilities to recognize and reverse overdoses (Green et al., 2008), but we hypothesize that the lack of a standardized or widely implemented course (e.g. like the American Heart Association’s Basic Life Support course) is a barrier.

When patients recalled that a naloxone prescription was given to them, it was filled nearly three-fourths of the time. We were interested to see that 8% of patients used the naloxone. About a third of the time, it was used on the patient, and the remainder of times it was used on someone else. A large distribution program in Norway found that 15% of high-risk individuals given naloxone used it (Madah-Amiri et al., 2019), and individuals who were treated by the health services arm of the Cook County Department of Corrections used dispensed nasal naloxone 38% of the time (Leung et al., 2021). Individuals who are “multiple overdose responders” (bystanders who have responded to two or more overdoses) are particularly important to equip with naloxone (Wagner et al., 2022). Our population of patients tends to be of lower acuity and many reside in rural areas that may not frequently encounter overdose victims as bystanders, but the rate of use was still notable.

Unfortunately, cost remains a barrier to obtaining naloxone. Thirty-six patients did not obtain the naloxone because it was too expensive. When insurance did not pay for the medicine, the median cost was $60. For these individuals, the over-the-counter version that was recently approved may reduce the burden (Harris, 2023), although the current price point of about $45 may still be too high for some people to obtain it (Becker, 2023). We were encouraged that most insured patients had no co-pay, and that those that did have co-pays reported a lower cost (median $10) than those who were uninsured. Only a few patients reported that the pharmacy did not have naloxone, which is a marked improvement over previous research from 2020 to 2021 that found that over 30% of studied pharmacies did not carry naloxone (Hill et al., 2022). The increase in community-based distribution of free naloxone over the past several years, including via vending machines, is a promising modality to reduce costs (Wagner et al., 2022).

About a third of patients who did not fill the prescription for naloxone stated they already had it on hand. Conversely, about 36% of patients who did not fill it said they did not believe it was needed. For these patients, education is essential as return to use is a possibility with OUD, and even when not the case for the patient, dispensation of naloxone to people at risk for overdose, social service agency staff, family, and friends of people who use opioids is associated with decreases in overdose deaths at the community level (Walley et al., 2013). As a future step for our telehealth practice, we can encourage all providers to educate patients about naloxone and also consider the creation of online educational modules.

Finally, we were interested in learning which patient characteristics were associated with being more likely to fill the naloxone prescription. We did not find any differences in gender, although increased odds for female gender compared to male nearly reached statistical significance. This finding warrants further investigation, especially since the rate of overdose deaths is much higher in male individuals (Butelman et al., 2023). We also did not find significant differences in race and ethnicity, though this result may be due to the small number of non-white and Hispanic survey respondents. Knowing how to administer naloxone was associated with more than double the odds of filling the prescription, which again reiterates the importance of education about naloxone especially for at-risk individuals. Even a brief, 5–10 minute training course appears to be sufficient to increase naloxone knowledge amongst people who use opioids (Behar et al., 2015).

These results lay the groundwork for future research. It would be useful to determine if the survey respondents had ever witnessed or experienced opioid overdose in the past, in order to gauge this population’s overall risk and ascertain if scaling up naloxone dispensation is cost effective. Learning where patients who already had naloxone received it would also be helpful, as it would possibly indicate the most effective ways of distributing the medication. Future work could also test knowledge about naloxone administration and identify gaps that specific training could address, as well as the price point at which the cost of naloxone does not become a barrier to filling the prescription.

### 4.1 Limitations

This study is subject to several limitations that must be considered. First, the study instrument was created by our study team but was not validated. All answers are based on patient self-report, and there was no confirmation of their responses. This limitation extends to the question of “do you know how to administer naloxone?”, in which we relied on patient self-report as opposed to verifying knowledge. Although there were over 700 responses, the response rate (5.4% of all patients active in treatment and 3.9% of all individuals who could have seen the recruitment message and study link) is low and may not be representative of the entire population. Likewise, we did not determine the treatment status of the respondents, nor their criminal justice or residential status, which may also be important covariates. Providers in the group are expected to discuss and prescribe naloxone at the first visit, but we were unable to confirm that this was done. Because the survey was anonymous, we were not able to compare the characteristics of survey participants vs. non-respondents. It was not confirmed that, when naloxone was used, it was the prescription that we had provided. The length in time of treatment is longer than the average of all patients, further reiterating that respondents may be different than the overall group of patients. Additionally, the population of individuals treated for OUD with telehealth may be different than those who are more vulnerable and of higher risk, such as those who are undomiciled or actively using opioids. Respondents were largely white and non-Hispanic, and the majority reported using prescription opioids prior to treatment (as opposed to a minority using heroin and/or fentanyl), which again may limit external validity.

## 5 Conclusions

When prescribed naloxone, patients treated in a telehealth treatment program fill the prescription 72% of the time, and when it is filled, 8% use the naloxone, which we interpret as a high rate of use that justifies the practice. Although 86% of respondents knew how to administer naloxone, further education could help those who do not, and all patients should be informed of the benefits of having naloxone on-hand. Prescription benefit cost policy changes (either better insurance coverage, lower copays or lower over-the-counter cost) may assist those who did not fill the prescription because it was too expensive.

## Contributors

All authors made valuable contributions to the manuscript. All authors contributed to the research design of the project. ENM and SGW performed data collection. BB and SGW performed the analysis. All authors contributed to the drafting of the manuscript. SGW takes responsibility for the overall work.

## Institutional review board

The study was approved by the Western-Copernicus Group (WCG) Institutional Review Board (study #20235330).

## Presentation

This research was presented at the American Society of Addiction Medicine Annual Meeting in Dallas, TX in April 2024.

## Funding

This research did not receive any specific grant from funding agencies in the public, commercial, or not-for-profit sectors.

## CRediT authorship contribution statement

**Scott G. Weiner:** Writing – review & editing, Writing – original draft, Supervision, Project administration, Methodology, Investigation, Formal analysis, Data curation, Conceptualization. **Barbara Burke:** Writing – review & editing, Formal analysis. **Brian Clear:** Writing – review & editing, Supervision, Methodology, Conceptualization. **Emily N Miller:** Writing – review & editing, Project administration, Investigation, Data curation.

## Conflict of interest

Outside of this work, Dr. Weiner is funded by 10.13039/100000002National Institutes of Health grants 5R01DA044167, 5R01HS026753 and 1R01DA058315. Dr. Weiner is also on the acute pain committee of Vertex Pharmaceuticals, Inc and scientific advisory board of Cessation Therapeutics, Inc. All authors are either current or former employees of Bicycle Health, Inc., and Dr. Clear and Ms. Burke also have equity interest.

## Declaration of Competing Interest

All authors were employed by Bicycle Health, Inc at the time of this research. Dr. Clear serves as an officer, and Dr. Clear and Ms. Burke have equity interest. To reduce bias, the research team, comprised of Ms. Burke, Ms. Miller and Dr. Weiner, worked largely independently of the rest of the group. The research team was supervised by Dr. Clear, who is the only company officer on this paper. Dr. Clear did not modify the study’s findings in any way but did provide input on the study design and approval of the final manuscript. Furthermore, no one else from Bicycle Health edited or reviewed this work beyond the four authors.

## Figures and Tables

**Table:** 




